# Transcription factors direct epigenetic reprogramming at specific loci in human cancers

**DOI:** 10.3389/fgene.2023.1234515

**Published:** 2023-10-09

**Authors:** Han Jiang, Guoxin Li

**Affiliations:** Department of General Surgery, Nanfang Hospital, The First School of Clinical Medicine, Southern Medical University, Guangzhou, Guangdong, China

**Keywords:** epigenetic reprogramming, oncology, transcription factors, histone modification, instrumental variable regression

## Abstract

The characterization of epigenetic changes during cancer development and progression led to notable insights regarding the roles of cancer-specific epigenetic reprogramming. Recent studies showed that transcription factors (TFs) are capable to regulate epigenetic reprogramming at specific loci in different cancer types through their DNA-binding activities. However, the causal association of dynamic histone modification change mediated by TFs is still not well elucidated. Here we evaluated the impacts of 636 transcription factor binding activities on histone modification in 24 cancer types. We performed Instrumental Variables analysis by using genetic lesions of TFs as our instrumental proxies, which previously discovered to be associated with histone mark activities. As a result, we showed a total of 6 EpiTFs as strong directors of epigenetic reprogramming of histone modification in cancers, which alters the molecular and clinical phenotypes of cancer. Together our findings highlight a causal mechanism driven by the TFs and genome-wide histone modification, which is relevant to multiple status of oncogenesis.

## Introduction

Epigenetic reprogramming refers to the alteration of dynamic epigenetic marks (like histone modifications, DNA methylation, chromatin remodeling and some non-coding RNA molecules) on post-translational levels at various promoter or enhancer elements. Above all, modifications of histone tails are essential to define distinct gene expression states and other chromatin-based processes both actively (H3K4me3, H3K4me1 and H3K27ac, *etc.*) and silently (e.g., H3K9me3 and H3K27me3). Mounting evidence suggests that epigenetic modification plays a role in the diverse clinical behavior of cancers ranging from slow-growing to aggressive tumors, and thus contributes to the progression of cancers (Wang et al., 2019; Baksh et al., 2020; Chi et al., 2020; Li et al., 2021). Although various types of cancer exhibit common phenotypic characteristics like uncontrolled growth and apoptosis resistance, the epigenetic changes leading to these features can differ significantly among different cancers, resulting in a considerable landscape of heterogeneity. By characterizing epigenetic modification during cancer development and progression, researchers gained notable insights into cancer-specific epigenetic reprogramming.

Epigenetic modification is restricted locally and globally with varying substrate specificities created hierarchical modification patterns from individual promoters to the entire chromosomes. Such epigenetic modification patterns are induced by a set of regulatory enzymes, many of which are known oncogenes (Kurdistani, 2007). In fact, epigenetic modification enzymes are specific - but not loci-specific - to certain chemical groups. Hence the epigenetic reprogramming in cancer shows some general tendencies (McClellan et al., 2023). For example, tumor genome tends to be demethylated, and hypomethylation occurs only in specific genes. Nevertheless, more studies demonstrate loci-specific epigenetic modifications in cancer. The landscape of tumor epigenetic modification can result from complex determinations such as germline variation, somatic evolution, immune check-point and treatment.

Notably, recent studies report that transcription factors (TFs) are involved in the regulation of epigenetic reprogramming by introducing epigenetic modifications at specific loci in different cancer types, which provides evidence for the formation of specific epigenetic reprogramming signatures (Kant et al., 2022). AR-co-factors (e.g., FOXA1 and HOXB13) bring about H3K4me3/H3K27me3 bivalent marks at neural lineage–associated genes in Myc-driven advanced prostate cancer (Berger et al., 2019). IGFBP4 results in an AKT-EZH2 reciprocal loop to drive H3K27me3-mediated epigenetic reprogramming in hepatocellular carcinoma (Lee et al., 2018). Still, the current knowledge of TFs as general regulators of epigenetic reprogramming is limited in certain genes and cancer types. It remains unclear how many TFs are involved in epigenetic reprogramming and how these TFs impact cancer epigenomes. To inform future functional study in cancer epigenetics, we systematically evaluated the impacts of 636 transcription factor binding activities on histone modification in 90 cell lines of 24 cancer types. We used mutations of TFs as instrumental proxies and regressed histone modification to the binding activity of TF. To this end, we identified 10 TFs-Histone markers pairs which strongly regulated the epigenome in cancers. Cooperative interaction among regulatory factors is often required to achieve precise regulation. We further focused on the co-binding localization at specific regulatory elements within the genome to achieve accurate regulation of target genes to investigate the impacts on the molecular and clinical phenotypes of cancer. Our findings suggested a causal mechanism for epigenetic reprogramming in cancer driven by the TFs, which helps better understand the process of tumorigenesis and treatment.

## Materials and methods

### Data collection

We collected ChIP-seq data processed from the Encyclopedia of DNA Elements Project (ENCODE, http://encodeproject.org) (Moore et al., 2020) for the human genome (hg38) including seven histone markers (H3K4me3, H3K36me3, H3K4me1, H3K27ac, H3K79me2, H3K9ac, H4K20me1) and 759 TFs. The MACS2 algorithm was used to identify the influence of genome complexity to evaluate the significance of enriched ChIP regions with narrow peaks mode (TFs) and broad peaks mode (histone modifications). We defined the binding activities of TFs and histone modifiers as normalized signal enrichment of genomic region peaks. For each cell line, more than three histone markers were candidate. For each epigenome, at least two replicates of the input experiment were candidate. After filtration, 4,206 paired BED4+6 files from 636 TFs to 7 histone markers involved 24 cancer types were included in the next analysis.

We got the somatic variants and expression information from the Cancer Cell Line Encyclopedia (CCLE, https://sites.broadinstitute.org/ccle/, v19q2) (Nusinow et al., 2020). We defined the positive mutation status as that non-silent somatic mutation in the protein coding region of a gene, or any mutation identified in a non-coding gene, including nonsense, missense, frameshif indels, splice site mutations, stop codon readthroughs, change of start codon, in-frame indels.

We downloaded The CERES scores of 320 cancer cell lines from The Cancer Dependency Map Project at Broad Institute (DepMap, https://depmap.org, v19Q2) (Barretina et al., 2019). The CERES score is an algorithm used to calculate the degree of gene dependency based on results from CRISPR-Cas9 essentiality screens. The score took into consideration the impact of gene copy number variations on the results. (Meyers et al., 2017). Data on the sensitivity of 305 drugs, measured by IC_50_, across 988 cancer cell lines, was obtained from the Genomics of Drug Sensitivity in Cancer database (GDSC; https://www.cancerrxgene.org).

### Generalized linear regression analyses suggest candidate TFs of epigenetic reprogramming

To estimate the linear effects of each TF on the epigenetic reprogramming, we performed Ordinary Least Squares (OLS) regression in order to infer the correlation between TFs binding activities and Histone marks binding activities in pan-cancer level. The regression coefficients of β represented the effect sizes of the binding activities of TFs. The regression coefficients of α represented the effect sizes of other covariates.

$${His}_{ki}∼{\beta_{ij}TF}_{ij}*C_{i}+\alpha_{i}\left(\begin{array}{c} E_{i}\\ T_{i} \end{array}\right)+\varepsilon_{ijk}$$

Here, εijk∼ N (0, σ2) was a Gaussian error; ${His}_{ki}$ referred to the binding activities of the *k*th Histone marks of *i*th cell lines; ${TF}_{ij}$ referred to the binding activities of *j*th TFs of *i*th cell lines. $E_{i}$ , $C_{i}$ and $T_{i}$ was the expression of TFs, classification of Histone marks (we set “activated” and “silent”) and the cancer types respectively. The regression coefficient α_i_ was a 1*2 vector responded to two covariates of E_i_ and T_i_. We corrected the multiple *p* values of the regression coefficients through “Benjamini–Hochberg” method, and we got the FDR for each TF. The significant TFs (FDR <0.1, lower than 10% of the false-positive rate associated with a *p*-value of 0.05 for each hypothesis testing) were candidate for the next analysis.

### Instrumental variable regression analysis

Instrumental variable analysis was used to figure out the driver TFs by performing the R package ivpack(v1.2), of which, their binding activities at the genetic loci resulted in the epigenetic reprogramming. Briefly, the dependent variable was the binding activities of histone marks (His), and the independent variable was the binding activities of TFs that was significantly associated with His, and the genetic instruments were the binary mutation status (wild type or mutated) of the TFs and the covariates above (the expression of TFs and the cancer types respectively).

$${His}_{ki}∼\beta_{0}+\beta_{1}{TF}_{ij}+E_{i}+T_{i}|{Mut}_{ij}+E_{i}+T_{i}+\varepsilon_{ijk}$$

Here, εijk∼ N (0, σ2) was a Gaussian error; ${His}_{ki}$ referred to the *k*th Histone marks of *i*th cell lines; ${TF}_{ij}$ referred to the binding activities of *j*th TFs of *i*th cell lines; ${Mut}_{ij}$ referred to the mutated status of *j*th TFs of *i*th cell lines. $E_{i}$ , and $T_{i}$ was the expression of TFs and the cancer types respectively. The IV models were estimated by Two-Stage least squares (2SLS). We corrected the multiple *p* values of $\beta_{1}$ through “Benjamini–Hochberg” method and got the FDR for each TF to evaluate the significance of the independent variables. To figure out the significance of the instrumental variables, we recalculated the *p* values for the weak instruments test using the Kleibergen-Paap rank Wald F-statistic and estimated the false discovery rate. (FDRweak) The significant TFs were considered as “driver TFs”. (FDR <0.1 and FDRweak <0.1).

### Identify the drug-related activities of EpiTFs in cancer cell lines

To evaluate the effects of the activities of EpiTFs on the drug sensitivity to therapy, we used a linear model to regress the mean mRNA expression of co-regulated genes for each TFs-HMs pair and the IC_50_ of drugs. According to the targets of drugs, we divided the drugs into different classes. Thus, we could calculate the OR values associated with different pathways.

$${IC50}_{ik}∼\beta_{0}+\beta_{1}{mRNA}_{ij}+C_{i}+\varepsilon_{ijk}$$

Here, εijk∼ N (0, σ2) was a Gaussian error; ${IC50}_{ik}$ referred to the *k*th drugs of *i*th cell lines; ${mRNA}_{ij}$ referred to the mean mRNA expression of co-regulated genes of *j*th TFs in *i*th cell lines.

### Gene sets enrichment analysis

To assess the overrepresentation of the target gene for each EpiTFs in established cancer gene sets, we used the R package GeneOverlap(v1.36.0) to conduct Fisher’s exact test. For the pathway enrichment analysis, we adopted the R package clusterProfiler(v4.8.1) to perform hypergeometric test in the target gene for each EpiTFs. The reference was hallmark gene sets (https://www.gseamsigdb.org/gsea/msigdb/collections.jsp).

### Survival analysis

A total of 3,295 EpiTFs related TCGA patients, including Prostate Cancer (PRAD, N = 494), Colon Cancer (COAD, N = 287), Breast Cancer (BRCA, N = 789), Lung Adenocarcinoma (LUAD, N = 509), Pancreatic Cancer (PAAD, N = 170), Liver Cancer (LIHC, N = 358), Kidney Clear Cell Carcinoma (KIRC, N = 366), Cervical Cancer (CESC, N = 286), Bile Duct Cancer (CHOL, N = 36), were included to analysis clinical outcome.

We employed the Kaplan-Meier approach to estimate the overall survival (OS), and evaluated the divergence between groups by conducting the log-rank test. *p* < 0.05 was considered significant.

### PPI network analysis

The search tool for retrieval of interacting genes (STRING, https://string-db.org) database (Szklarczyk et al., 2021) is served to predict the protein-protein interaction network (PPI network). To identify potential interactions between target genes according to different EpiTFs, R package STRINGdb(ver.2.10.1, database version = 11.5, species version = 9,606) was employed. Active interactions (score threshold = 700) were applied to construct the PPI networks. R packages igraph(ver.1.4.2) and ggraph(ver.2.1.0) were used to constructive and visualize the PPI network. In the networks, the nodes referred to the proteins and the edges represent the interactions. To display the highly connected regions of the network, we set the following criteria: minimum degree = 5, edge weights = combined score, the minimum average edge weight within the cluster = 0.05.

## Results

### TFs involved in epigenetic modification

**FIGURE 1:**
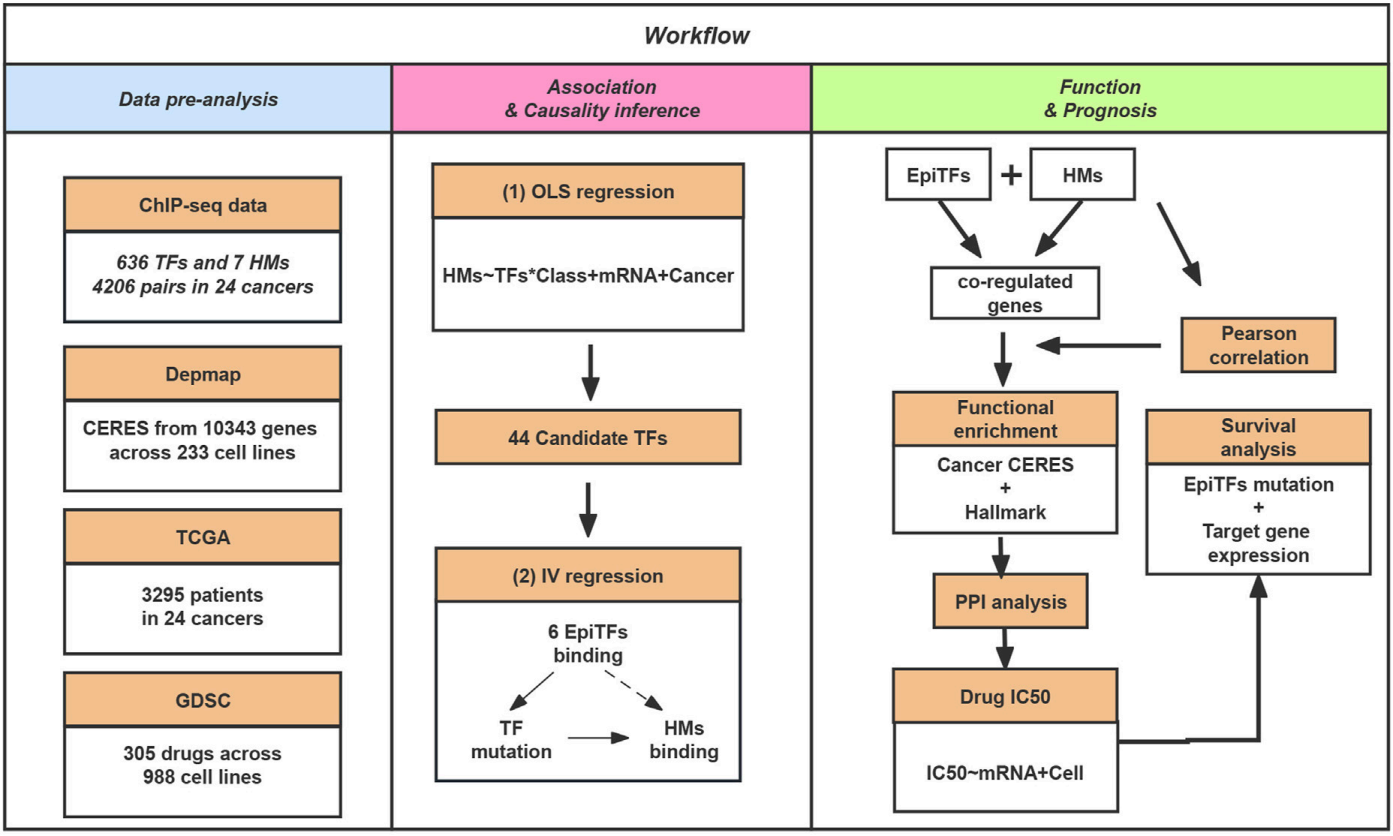
The schematic workflow of our study. In this study, we collected ChIP-seq data for TFs and HMs. Then, we inferred the causal biological mechanisms for the candidate determinants of the histone modification via EpiTFs mutation. In addition, we perform gene sets enrichment, PPI analysis, Drug resistance and Survival analysis for the co-regulated loci of EpiTFs and HMs to investigate the biological function and clinical outcome of EpiTFs.

To evaluate the dynamics of epigenetic modification in tumor genomes systematically and identify the reliable driver TFs, we collected ChIP-seq data for TFs and histone marks (HMs) from the ENCODE database. After a raw filtration (see Methods; Figure 1), the dataset included 636 transcription factors and 7 epigenetic marks (H3K4me3, H3K36me3, H3K4me1, H3K27ac, H3K79me2, H3K9ac, H4K20me1) from 54 cell lines among 17 different tumors. Then we performed an Ordinary least squares (OLS) regression model to screen for TFs and their corresponding epigenetic marks with consistent binding activities at their overlapped loci. To our surprise, we only obtained 6.9% (N = 44) candidate TFs (FDR <0.1), which can be considered to be associated with HMs, suggesting a finite correlation between TFs and epigenetic changes.

In order to better understand the causal relationship between TFs and epigenetic changes, we further generated an instrumental variable (IV) regression for the 44 candidate driver TFs based on a mechanism of epigenetic reprogramming: the mutational status of a candidate TF directly alter its binding activity in cancers and then influence the epigenetic changes (Figure 1). We used the mutational status of the TFs as the instrumental variable to exogenously separate the changes in explanatory variable that are independent of the error term and ignore the part of explanatory variable that causes bias in the OLS estimator. This change could be considered as the causal effect in TF binding strength on corresponding tumor genomic HMs. As a result, we identified 10 significant TFs-HMs pairs that satisfied the condition of the mutational statuses of TFs being an exogenous variable (FDR <0.1 and FDRweak <0.1), among which 6 TFs (AR, EP300, FOXA1, GATA3, POLR2A, TP53) and 3 HMs (H3K4me1, H3K4me3, H3K27ac) were included (Table 1). Of note, H3K4me3 was considered as a marker of promoter regions, and H3K4me1 and H3K27ac were markers of enhancer regions. Thus, all of our driver TFs mainly impacted the promoter and enhancer elements: mutations in TFs such as AR, EP300, and TP53 tended to affect the enhancer regions of downstream target genes, while other transcription factors affected the binding intensity of both promoter and enhancer regions (Table 1).

**TABLE 1:** The results of IV analysis for 10 EpiTFs-HMs pairs.

TFs	HMs	*p*-value	OR	Waldtest	Weak instruments	Wu_Hausman
AR	H3K4me1	0.002	0.653	0.000	0.000	0.000
EP300	H3K4me1	0.000	1.057	0.000	0.000	0.142
EP300	H3K27ac	0.008	0.810	0.000	0.000	0.025
FOXA1	H3K4me3	0.000	1.879	0.000	0.000	0.000
FOXA1	H3K4me1	0.000	1.314	0.000	0.000	0.000
GATA3	H3K4me3	0.000	22.609	0.000	0.000	0.000
GATA3	H3K4me1	0.000	1.991	0.000	0.000	0.003
GATA3	H3K27ac	0.003	2.593	0.000	0.000	0.000
POLR2A	H3K4me3	0.000	0.832	0.000	0.000	0.000
TP53	H3K4me1	0.000	3.340	0.000	0.000	0.000

### EpiTFs influence specific cancer pathways

As the epigenetic determinants of driver TFs (also we named it EpiTFs) influence many biological processes in tumors, we next explored how the EpiTFs impact carcinogenesis and the consequent biological–clinical characteristics of cancer.

**FIGURE 2:**
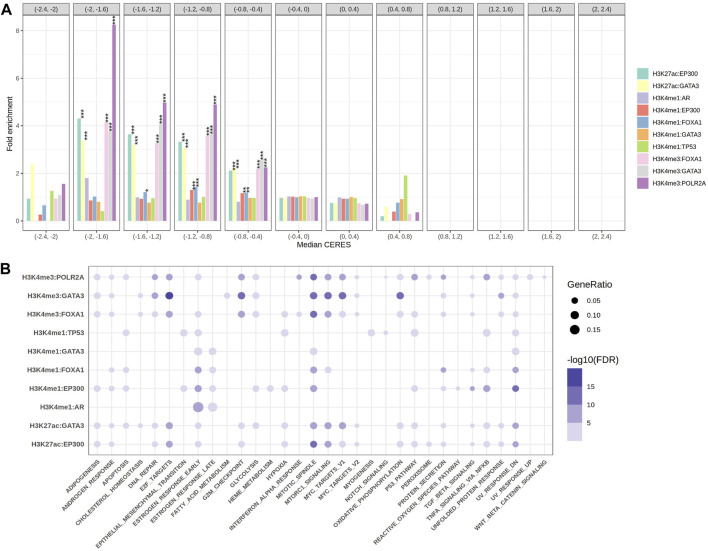
The gene sets enrichment analysis. **(A)** The grouped bar plot showed the fold enrichment results of the target genes come from 10 EpiTFs-HMs pairs in different groups of cancer dependency gene sets (the length of each intervals was 0.4 and CERES <1 meant cancer dependency). **(B)** The bubble chart showed the 50 hallmark gene sets enrichment results of the target genes come from 10 EpiTFs-HMs pairs (the color indicated FDR, the size of the dots indicated gene ratio).

Firstly, we obtained the downstream target genes through figuring out the overlapped regions for each TFs-HMs pair. The most recent advances in gene-editing technology has allowed for precise examination of the role of specific genes in driving the growth of cancer cells. According to the CERES scores for genes from Depmap portal, we categorized 10,343 genes of cancer dependency according to the median CERES score across 233 cell lines. We then compared the fold enrichment of our target genes among 10 TFs-HMs pairs. We found out that almost all the target genes of these pairs were significantly enriched as the oncogenes, whose CERES score is less than zero (CERES = −2 ∼ −0.4, Figure 2A). Among them, the target genes of H3K4me3 resulting from FOXA1, GATA3 and PLOR2A as well as H3K27ac resulting from EP300 and GATA3 were significantly enriched in the gene sets (CERES = −2∼−0.4, *p* < 0.001), signifying a broad dependency for tumor proliferation. Compared to H3K4me3 and H3K27ac, H3K4me1 target genes were weaker to cancer dependency *in vitro* (Figure 2A).

Secondly, to better know the biological characteristics of these TFs-HMs pairs, we performed pathway enrichment analysis on their target genes using the Hallmark reference databases. The results showed that the target genes of AR:H3K4me1 were mainly enriched in the estrogen-ER activity, and the target genes of TP53:H3K4me1 were enriched in pathways related to hypoxia, apoptosis, UV, and the p53 pathway (Figure 2B), which is consistent with our understanding of these two transcription factors (Mills, 2014; Levine, 2020). It is worth noting that FOXA1 target genes were mainly enriched in pathways related to apoptosis, mitosis, mTOR, TNF-α (Figure 2B), indicating its possible involvement in tumor malignant proliferation and supporting our fold enrichment of CERES scores above (Figure 2A).

### EpiTFs define specific subtype of cancer

**FIGURE 3:**
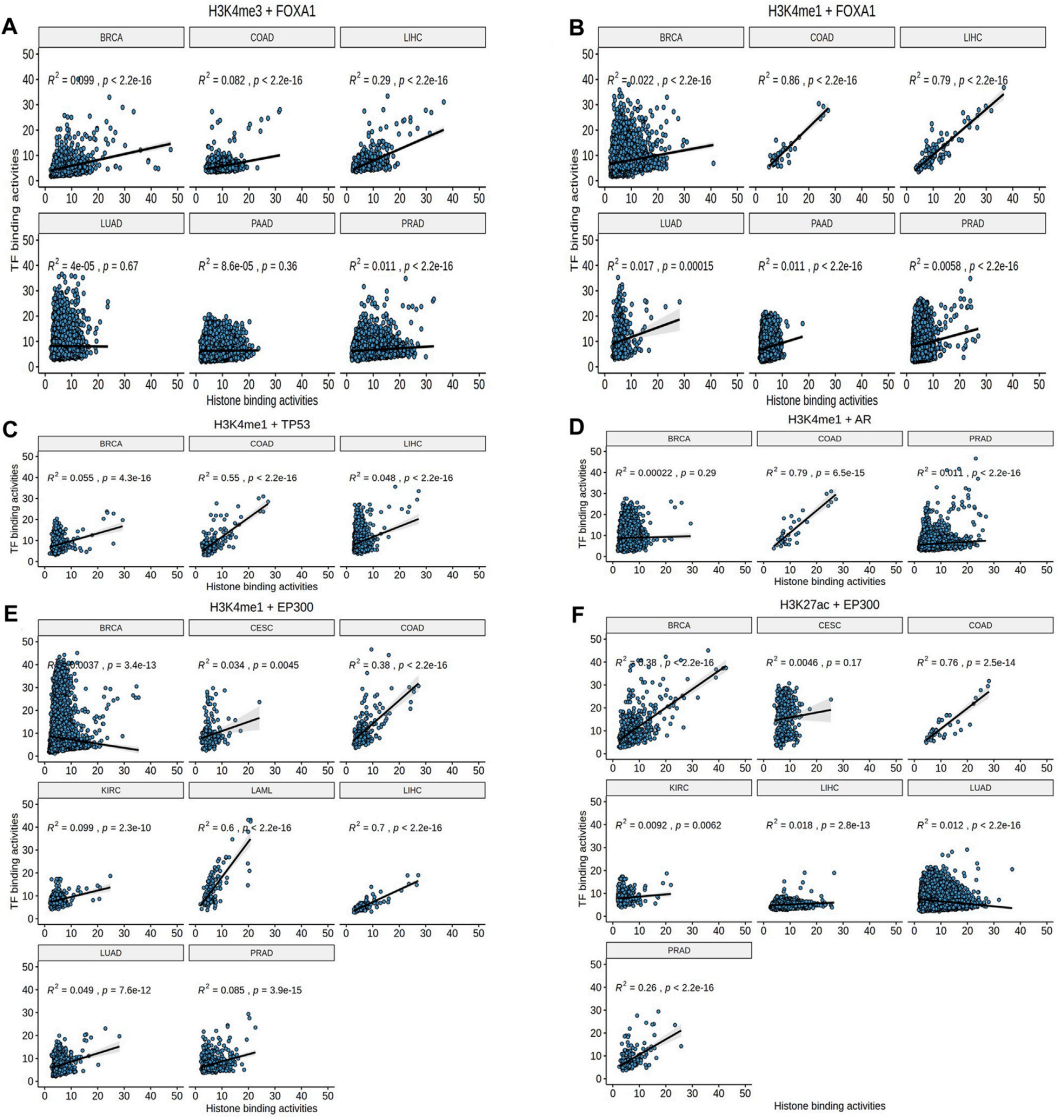
The Pearson correlation analysis. **(A, B)** H3K4me1 rather than H3K4me3 showed consistent effects (Pearson correlation) in FOXA1. **(C–F)** TP53, AR, and EP300 had significant correlations in COAD.

To further validate whether the causal relationship among these 10 pairs define specific subtype of cancer, we plotted the binding activities at the overlapped loci of EpiTFs and HMs in different tumors, and calculated the Pearson correlation coefficient between them. We observed that for FOXA1, it was more closely associated with the enhancer marker H3K4me1 than the promoter marker H3K4me3. The FOXA1:H3K4me1 correlation coefficient was 0.86, 0.79, while the FOXA1:H3K4me3 coefficient was only 0.082, 0.29 in COAD and LIHC respectively (Figures 3A,B), indicating that FOXA1 is enhancer-dependent transcription factor in COAD and LIHC. In addition to FOXA1, we also found that three other transcription factors - TP53, AR, and EP300 - still had significant correlations in COAD (correlation coefficient *R* ^2^ > 0.3), including TP53:H3K4me1 (*R* ^2^ = 0.5), AR:H3K4me1 (*R* ^2^ = 0.79), EP300:H3K4m1 (*R* ^2^ = 0.38), and EP300:H3K27ac (*R* ^2^ = 0.55) (Figures 3C–F). It suggested that these TFs were closely related to tumors in COAD. Interestingly, the corresponding epigenetic markers of these TFs were all enhancer markers such as H3K4me1 and H3K27ac (Figures 3B–F). Therefore, we believed that the epigenetic changes mediated by these TFs mainly concentrated on the enhancer regions of the genome. However, GATA3 (*R* ^2^≤0.3, Supplementary Figures S1B–D) and PLOR2A (*R* ^2^≤0.3, Supplementary Figure S1A) led less correlation to epigenetic changes.

Additionally, some TFs-HMs pairs were occurred in specific tumor. For example, only EP300 and H3K27ac were closely related to BRCA (*R* ^2^ = 0.47, *p* < 2.2e-10, Figure 3F); FOXA1 and EP300 were both related to H3K4me1 in LIHC (*R* ^2^ = 0.79, *p* < 2.2e-16; *R* ^2^ = 0.7, *p* < 2.2e-16, respectively, Figures 3B,E). These prominent pairs in specific tumors may reflect the epigenetic subtypes as well as epigenetic features for the tumor biology.

### EpiTF activities are associated with treatment responses

**FIGURE 4:**
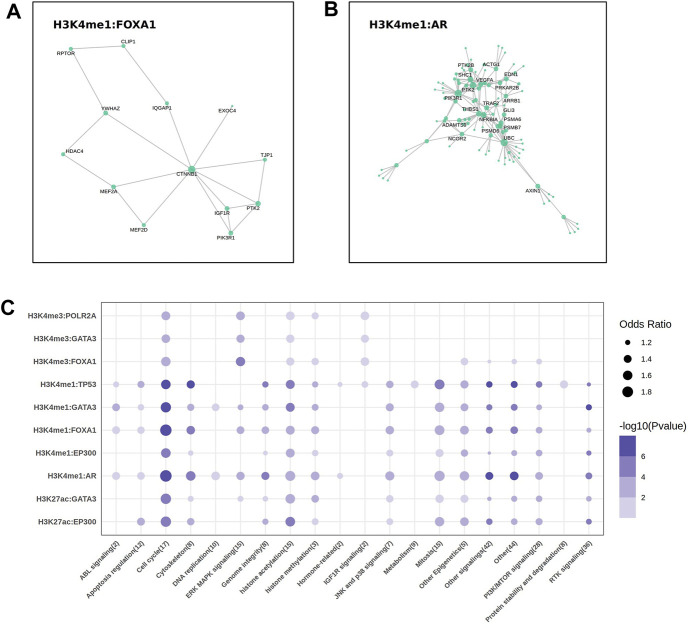
The key molecular and drug sensitivities analysis. **(A, B)** The protein-protein interaction (PPI) analysis showed the labeled hub protein (degree >5) in FOXA1 (A) and AR (B) regulatory network. **(C)** The bubble plot showed the correlation between the sensitivities of 302 drugs targeted to different pathways and the mean expression of the target genes come from 10 EpiTFs-HMs pairs.

To better describe the biological function of these regulatory regions on genes, we generated a protein-protein interaction (PPI) network analysis on the EpiTFs and epigenetic marks. In PPI networks, “hub nodes” usually refer to nodes with extremely high degree. Researchers often focus on these “hub nodes”to gain deeper insights into the topology of PPI networks and related biological processes (Wang et al., 2023). We labeled the hub proteins that were associated with at least 5 other genes. As a result, the key proteins in the Wnt pathway, such as CTNNB1 (β-Catenin) and MEF2A/D (MEF2), which were influenced by FOXA1:H3K4me3, were located in the core position of the interaction network (Figure 4A). Similarly, among the target genes affected by AR:H3K4me1, we also spotted the presence of key proteins in the Wnt-β-Catenin pathway such as AXIN1, as well as targets of the PI3K/NF-κB pathway (Figure 4B), which respond to cell proliferation. HDAC4, notably, exists in the network, which may be an important auxiliary factor for FOXA1 to participate in epigenetic modifications.

The expression of genes co-regulated by EpiTFs and HMs reflected the biological function levels of EpiTFs. To assess the therapeutic implications of the EpiTFs activities, we used a linear model to calculate the association between the mean expression of the co-regulated genes and the IC_50_ in 304 clinical small-molecule agents. As a result, four EpiTFs (TP53, AR, EP300 and FOXA1), which were strongly corelated with HMs derived a strong resistance to the similar agents (OR > 1 and *p* < 0.001), which targeted to the cell proliferation such as cell cycle, mitosis, and PI3K/MTOR signaling (Figure 4C). Notably, chromatin histone acetylation was also related to higher activities of these four EpiTFs in H3K4me1, H3K27ac (Figure 4C), suggesting that HDAC inhibitors may intervene in the development of corresponding tumors. This requires further verification in subsequent research. Moreover, the activities induced by H3K4me3 showed weaker drug effects than those induced by H3K4me1 and H3K27ac (Figure 4C), demonstrated that EpiTFs-driven epigenetic reprogramming tended to occur in enhancer elements rather than promoter elements when suffered from drug resistance.

### EpiTFs are associated with clinical outcomes

**FIGURE 5:**
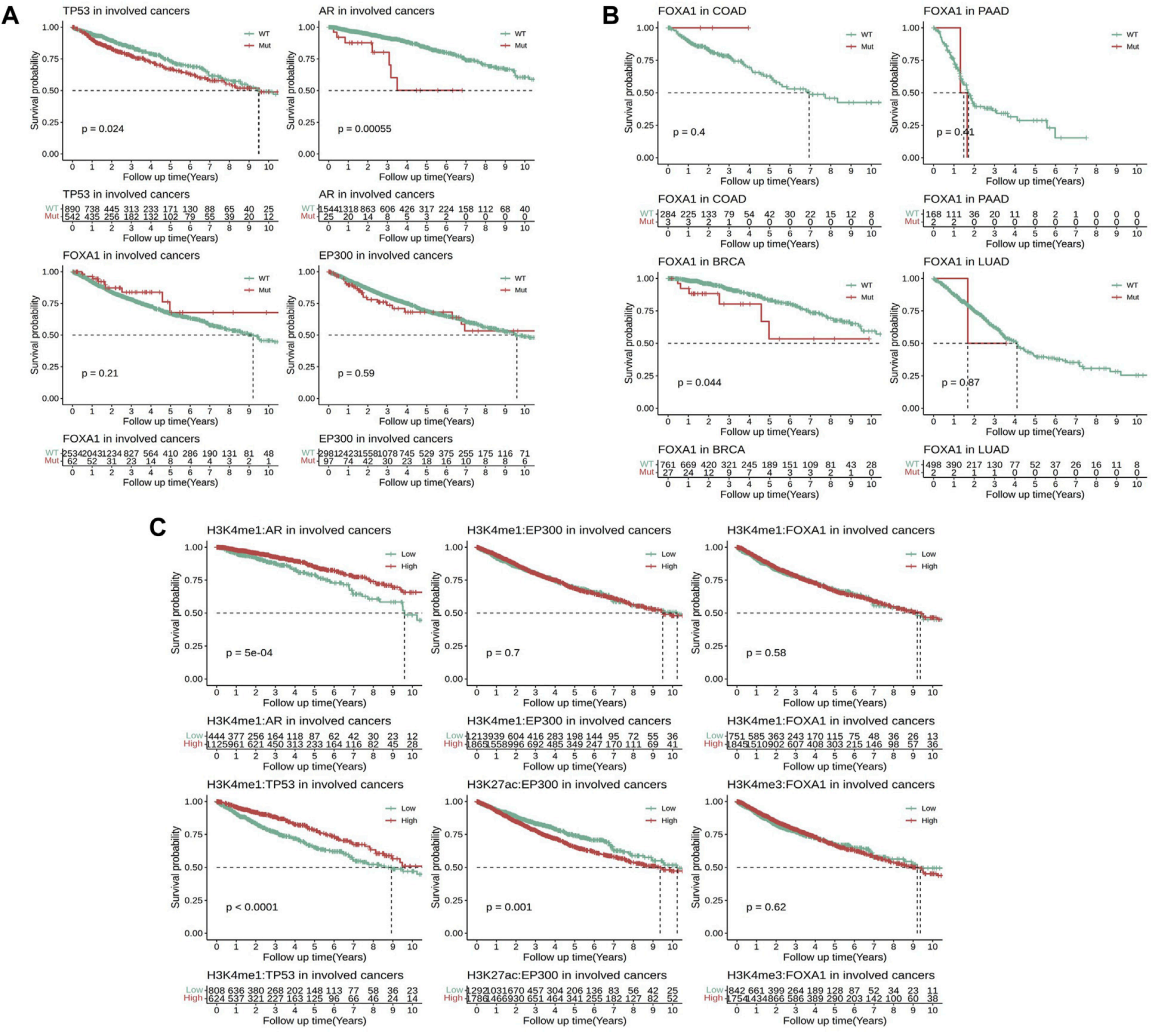
Comparison of survival analysis between TCGA patients with different mutation statuses and transcriptional activities in four activated EpiTFs. **(A)** Kaplan‒Meier curves for patients with different mutation statuses in four activated EpiTFs in pan-cancer level. **(B)** Kaplan‒Meier curves for patients with different mutation statuses of FOXA1 in specific cancers. **(C).** Kaplan‒Meier curves for patients with different transcriptional activities in 6 Epi-HMs pairs at pan-cancer level.

To further clarify the impact of these EpiTFs on prognosis of clinical outcome, a total of 3295 TCGA patients were enrolled to investigate the effects of different mutation statuses and target gene expression on overall survival. The results indicated that among the four activated EpiTFs, AR mutation and TP53 mutation led to a poor prognosis in the EpiTFs positive cancers (*p* = 5.5e-4 and *p* = 0.024, Figure 5A). The 5-year OS were 50.2% (95% CI 28–89.9) and 67.05% (95% CI 64.33–69.85) respectively. While FOXA1 and EP300 had no significant effect on patient survival in all the involved tumors (*p* = 0.21 and *p* = 0.59, Figure 5A). In comparison to different types of tumors, FOXA1 mutation in BRCA (27/788 mutated) resulted in worse prognosis (*p* = 0.044, the 5-year OS was 53.5%, 95% CI 35–58.7, Figure 5B); COAD patients (3/287 mutated) with FOXA1 mutation had better prognosis but without statistically significance (*p* = 0.4, Figure 5B). Conversely, EP300 mutation did not affect the prognosis of overall survival at both pan-cancer level and cancer specific level (Figure 5A, Supplementary Figure S2C). Next, we grouped patients by the median mRNA expression of these EpiTFs target genes. We found that patients with lower co-regulated target genes expression of AR as well as TP53 had a worse prognosis (P = 5e-4 and P = 1e-04, Figure 5C). And both of them were associated with H3K4me1 modification (The 5-year OS rates of H3K4me1:AR and H3K4me1:TP53 were 79.12% (95% CI 73.66–85) and 65.09% (95% CI 60.44–70.1), respectively). To our surprise, the higher expression of target genes regulated by EP300:H3K27ac (the 5-year OS was 65.57%, 95% CI 62.44–68.84) rather than EP300:H3K4me1 led to a poorer prognosis (*p* = 0.001, Figure 5C).

## Discussion

In this study, we aimed to evaluate the dynamics of epigenetic modification systematically and identify the reliable driver TFs in human cancers. In traditional multiple linear regression models, if there is a correlation between some independent variables and the error term, it will lead to biased OLS estimates, of which the obtained regression coefficients loss a causal interpretation (Lu et al., 2023). To eliminate endogeneity problems and obtain accurate estimates of causal effects, we used the IV regression to infer the significant causal relationship between TFs and HMs. We identified a total of 6 EpiTFs (FOXA1, AR, EP300, TP53, GATA3, LOR2A), which resulted in the corresponding histone modification (e.g., H3K4me3, H3K4me1 and H3K27ac) (Table. 1). Among them, FOXA1 was wildly regarded as pioneer transcription factor (pTF) in prostate cancers. Recent studies demonstrated that pTFs referred to a type of TFs that can bind to packed chromatin tightly and promote the opening and accessibility of gene promoter regions and even enhancer regions (Iwafuchi-Doi et al., 2016; Parolia et al., 2019). Similarly, the EpiTFs mainly caused H3K4me1 and H3K4me3 in our study (Table. 1), suggesting that both of EpiTFs and pTFs fulfill their functions through Cis- and trans-regulated. Moreover, pTF FOXA1 usually collaborated with other partners (like AR) to mediate processes such as histone modification and chromatin remodeling, enabling cells to respond rapidly to external stimuli and achieve complex regulatory networks (Barbieri et al., 2012). Thus, we supposed that the EpiTFs was an extensive concept including pTFs and their partners. However, since bulk TFs lacked their ChIP-seq data (86.25%, 276/320), it was unable to identify all the EpiTFs in human cancers. Increased sample size can improve the statistical power to identify more EpiTFs in the future study.

After further confirmed with the binding activities between TFs and HMs, 4 of 6 EpiTFs (FOXA1, AR, TP53, EP300) were thought to be more positive to induce corresponding histone modification in one or multiple tumors (Figure 3). Among them, EP300 was an acetyltransferase that directly catalyzed H3K27 acetylation modification and promoted chromatin relaxation and transcriptional initiation (Cui et al., 2023), which was in line with the modes of EpiTFs. Through comparing the CERES fold enrichment of our target genes among 10 TFs-HMs pairs, we found that the target genes of these EpiTFs were significantly enriched as cancer dependent genes. The target genes of FOXA1 and H3K4me1 were significantly enriched in both the negative interval (CERES = -1.6∼-0.4, *p* < 0.001) (Figure 3A). Indeed, it is still unknown how FOXA1 alterations affect the prognosis in human cancers, and how FOXA1 is able to serve as both tumor-suppressor (Jin et al., 2013; Jin et al., 2014; Song et al., 2019) and oncogenic genes (Robinson et al., 2011; Pomerantz et al., 2015). Consistent with this, FOXA1 mutations and transcriptional activities had no significant effect on overall survival in pan-cancer level (Figures 5A,C), and FOXA1 mutations only led to a worse prognosis in BRCA (*p* = 0.044, Figure 5B). FOXA1 mutations dysregulated estrogen-ER activity (Figure 2B) and were associated with worse outcome for metastatic ER + breast cancer (Hurtado et al., 2011; Arruabarrena-Aristorena et al., 2020). However, large breast cancer gene expression datasets revealed that histone acetyltransferases EP300 was correlated with the cancer stem cells and poor prognosis in triple negative breast cancer and basal-like Breast cancers (Ring et al., 2020). Compared to FOXA1, EP300 has higher association with H3K27ac in BRCA (R = 0.38, *p* < 0.001, Figure 3F), which could well support those viewpoints. Thus, TFs-HMs pairs characterized some biological features of tumors, and EpiTFs could define different subtype of tumors through their downstream histone modification.

In the PPI networks analysis, the results indicated that some key proteins (β-Catenin, MEF2, AXIN1) in the Wnt pathway were “hub nodes” in the interaction network of FOXA1:H3K4me1 and AR:H3K4me1 (Figures 4B,C). β-Catenin acted as an adhesion protein and accumulated in the nucleus when the Wnt signal was upregulated. As a coactivator of the TCF/LEF family of transcription factors, it can activate Wnt response genes, such as the genes encoding cell cycle proteins like cyclin-D and c-myc that promote cell proliferation, leading to tumor fast progression in cancers such as colon, ovarian, prostate, hepatoblastoma, and hepatocellular carcinoma (O'Brien et al., 2023). We assumed that both FOXA1 and AR resulted in the elevated H3K4me1 level, activating the Wnt/β-Catenin pathway to accelerate the process of downstream cell cycle and mitotic (Figures 2B, 4B). Moreover, HDAC4 was also the “hub node” occurred in the FOXA1 network (Figure 2B). It was reported that FOXA1 could be modulated by HDAC3 through the Wnt/β-catenin signaling in ovarian carcinoma (Lou et al., 2022). HDAC3 and HDAC4 are both histone deacetylases (HDACs), which participate in histone modification within the cell nucleus, altering the structure and function of chromatin by removing acetyl groups (Mustafa and Krämer, 2023). Despite these two HDACs belong to different classes (HDAC3 was HDACI, HDAC4 was HDACIIa), both of them were Zn2^+^ dependent HDACs and targeted by the pan-HDAC or HDACI/II inhibitors.

Regarding to TP53 and AR, the mutation of these two EpiTFs led to poor prognosis in involved patients (Figure 5A). Meanwhile, the diminished target gene expression of these two EpiTFs also resulted in a worse overall survival (Figure 5C). These results suggesting that the mutation of TP53 and AR caused the reduced binding of DNA. Indeed, the majority mutations in these 2 TFs resulted in defective gene function, including loss-of-function and gain-of-function gene alterations (Rebello et al., 2021; Soussi, 2022). However, in EP300, high transcriptional activities rather than genomic lesions were contributing to tumor progression at pan-cancer level (Figures 5A,C; Supplementary Figure S2C). In cancer cells, structural variations (SVs) in genome can disrupt three-dimensional chromosomal organization, so that the increased deposition of H3K27 (“enhancer hijacking”) promoted oncogene expression (Sungalee et al., 2021; Wang et al., 2021). Although mutated EP300 failed to convert the overall survival at pan-cancer level, high transcriptional activities in EP300:H3K27ac (rather than EP300:H3K4me1) target genes resulted in a worse prognosis (Figures 5A,C). It suggested that H3K27ac tend to aggravate the cancer biology of EP300 mutations though other mechanisms like enhancer-hijacking.

In summary, the study provides valuable insights into the role of EpiTFs in epigenetic modification and their association with cancer. We identified 6 driver EpiTFs via a causality inference including four strong EpiTFs (FOXA1, AR, TP53, EP300). They tended to induce histone modification in enhancer regions (H3K4me1 and H3K27ac). The downstream target genes of them were cancer dependent and enriched in some pathways related to cell proliferation as a whole. Of note, HDAC4 and Wnt/β-Catenin were played critical roles on FOXA1 and AR. These findings may have implications for the development of targeted therapies for cancer treatment.

## Data Availability

The original contributions presented in the study are included in the article/Supplementary Material, further inquiries can be directed to the corresponding authors.
