# Supplementary figures and images for: Transcription factors direct epigenetic reprogramming at specific loci in human cancers
Source: Front Genet. 2023 Oct 9;14:1234515. doi: 10.3389/fgene.2023.1234515 (PMC10591108; doi:10.3389/fgene.2023.1234515)

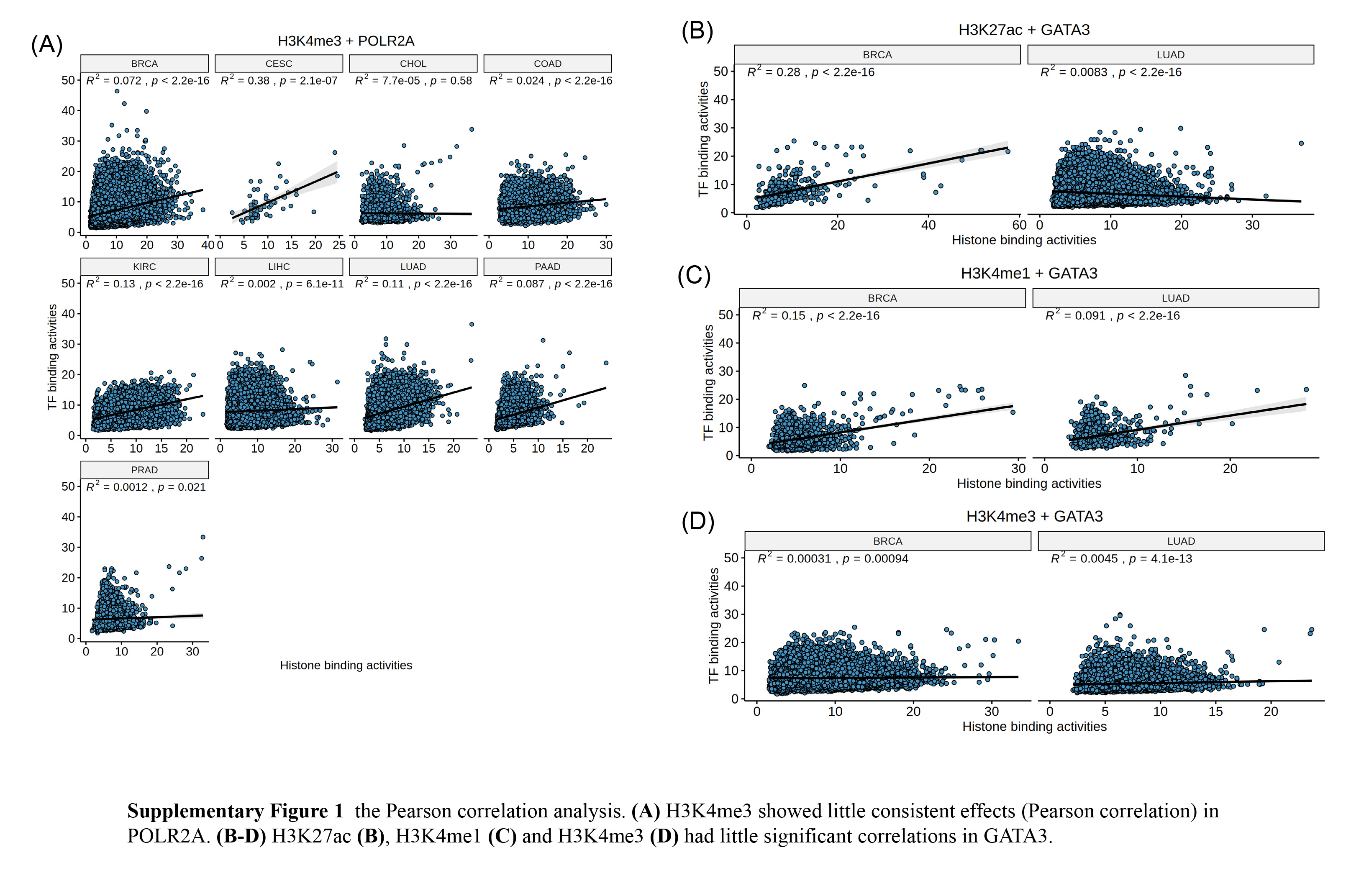

Supplement: Supplementary file 1 [file Image1.JPEG]

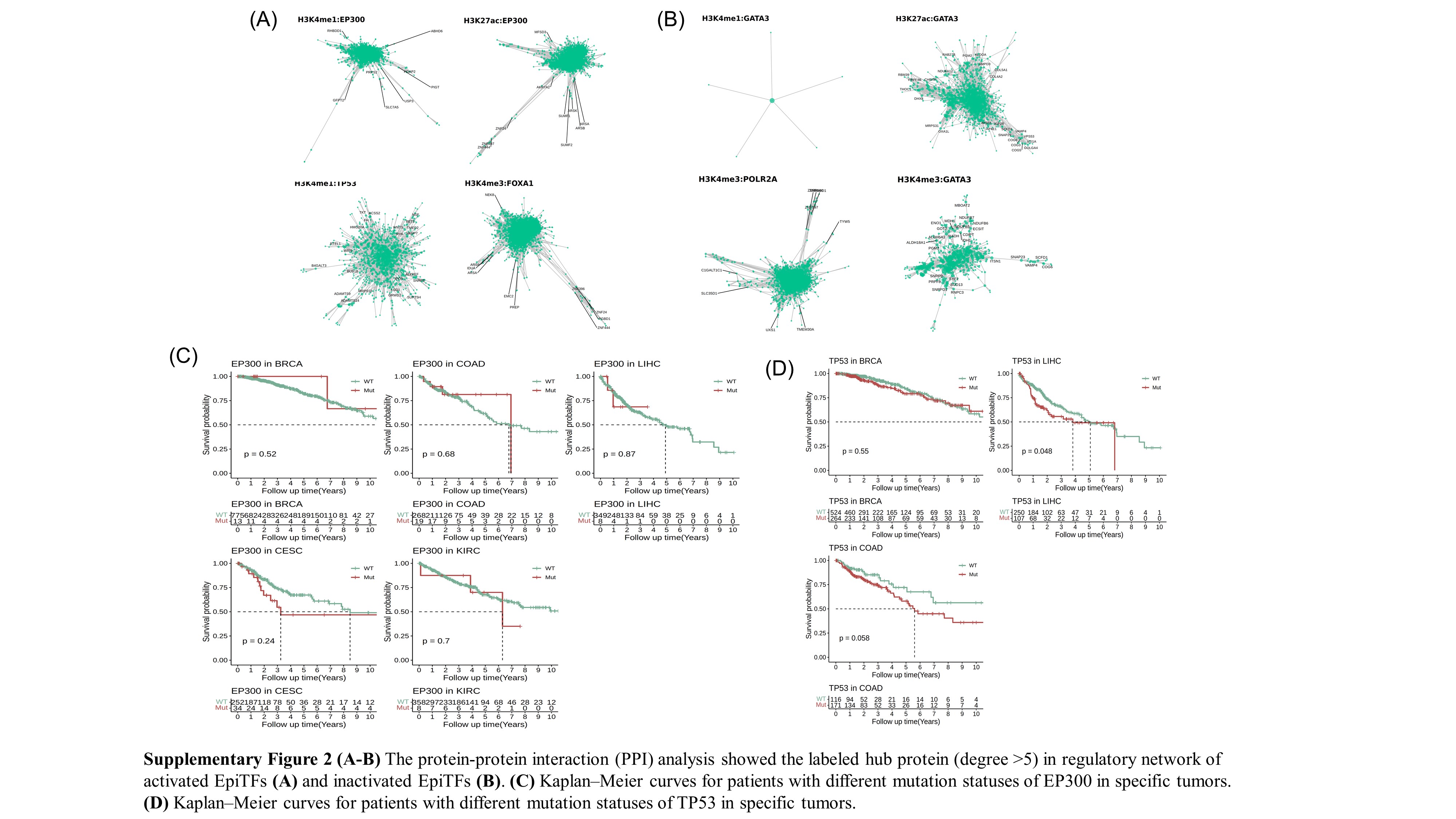

Supplement: Supplementary file 2 [file Image2.JPEG]
